# From Naturally-Sourced Protease Inhibitors to New Treatments for Fungal Infections

**DOI:** 10.3390/jof7121016

**Published:** 2021-11-27

**Authors:** Davier Gutierrez-Gongora, Jennifer Geddes-McAlister

**Affiliations:** 1 The Department of Molecular and Cellular Biology, University of Guelph, Guelph, ON N1G 2W1, Canada; davier@uoguelph.ca; 2 Centro de Estudio de Proteínas, Facultad de Biología, Universidad de La Habana, La Habana 10400, Cuba; 3 Canadian Proteomics and Artificial Intelligence Research and Training Consortium, Guelph, ON N1G 2W1, Canada

**Keywords:** proteases, protease inhibitors, fungal pathogens, natural compounds, biomedical applications, antimicrobial resistance

## Abstract

Proteases are involved in a broad range of physiological processes, including host invasion by fungal pathogens, and enzymatic inhibition is a key molecular mechanism controlling proteolytic activity. Importantly, inhibitors from natural or synthetic sources have demonstrated applications in biochemistry, biotechnology, and biomedicine. However, the need to discover new reservoirs of these inhibitory molecules with improved efficacy and target range has been underscored by recent protease characterization related to infection and antimicrobial resistance. In this regard, naturally-sourced inhibitors show promise for application in diverse biological systems due to high stability at physiological conditions and low cytotoxicity. Moreover, natural sources (e.g., plants, invertebrates, and microbes) provide a large reservoir of undiscovered and/or uncharacterized bioactive molecules involved in host defense against predators and pathogens. In this Review, we highlight discoveries of protease inhibitors from environmental sources, propose new opportunities for assessment of antifungal activity, and discuss novel applications to combat biomedically-relevant fungal diseases with in vivo and clinical purpose.

## 1. Introduction

Proteases hydrolyze the peptide bonds of polypeptides and proteins, with proteases accounting for 6% of total proteins in the human genome and 1–5% of microbial (e.g., bacteria, fungi, and virus) genomes [1]. Proteases are used by microorganisms in many processes, including stress response, nutrient acquisition, and protein maturation for cell division. Likewise, pathogens use these enzymes as important virulence factors in both direct and indirect damage of the host to: (i) gain access to nutrients [2]; (ii) destroy host cells and tissues to facilitate invasion and dissemination [3,4]; (iii) degrade host immune molecules for defense evasion [5,6,7]; (iv) promote pathogen propagation and maturation [8]; and (v) process self-molecules for pathogenicity [9,10]. Such roles promote the development of protease-based therapies [11] for pathogen-related diseases, including fungal meningitis [12], HIV/AIDS [13], candidiasis [14], aspergillosis [15], and COVID-19 [16].

Conversely, inhibition is one of the main molecular control mechanisms regulating proteolytic activity by which organisms use protease inhibitors to prevent self-damage [17], and provide protection against pathogens [18,19,20,21] or predators [22,23]. Currently, there are several protease inhibitors on the market for the management of human diseases, such as dabigatran and angiotensin converting enzyme inhibitors (ACEI) for the management of pulmonary embolism and hypertension, respectively [24,25]. Similarly, there are pharmaceuticals, such as bortezomib (clinically approved for the treatment of multiple myeloma by inhibition of proteasome complex) [26] with potential for applications against fungal pathogens. For example, in the widespread human fungal pathogen, *Cryptococcus neoformans* through regulation of virulence factor elaboration (i.e., polysaccharide capsule) [27,28]. However, such synthetic protease inhibitors can be plagued by low stability, high toxicity effects, or encounter resistance mechanisms, supporting the discovery of novel protease inhibitors from the natural environment [26,29]. Investigation of naturally-sourced protease inhibitors therefore, presents an alternative opportunity to expand our repertoire of antimicrobial agents and avoid such undesired features. 

In this Review, we highlight the role of proteases related to fungal virulence and the impact of protease inhibition as an anti-virulence strategy. Next, we argue the benefits of naturally-derived protease inhibitors through presentation of representative examples derived from plants, invertebrates, and microbial sources with a focus on antifungal activity. Finally, we propose opportunities to expand our current repertoire of antifungals through discovery and characterization of naturally-sourced protease inhibitors with potential applications in emergent diseases. The goal is to aid researchers in finding effective strategies with greater target specificity that are less prone to the evolution of resistance.

## 2. Protease Inhibition Exerts Anti-Virulence Effects on Fungal Pathogens

**Figure 1:**
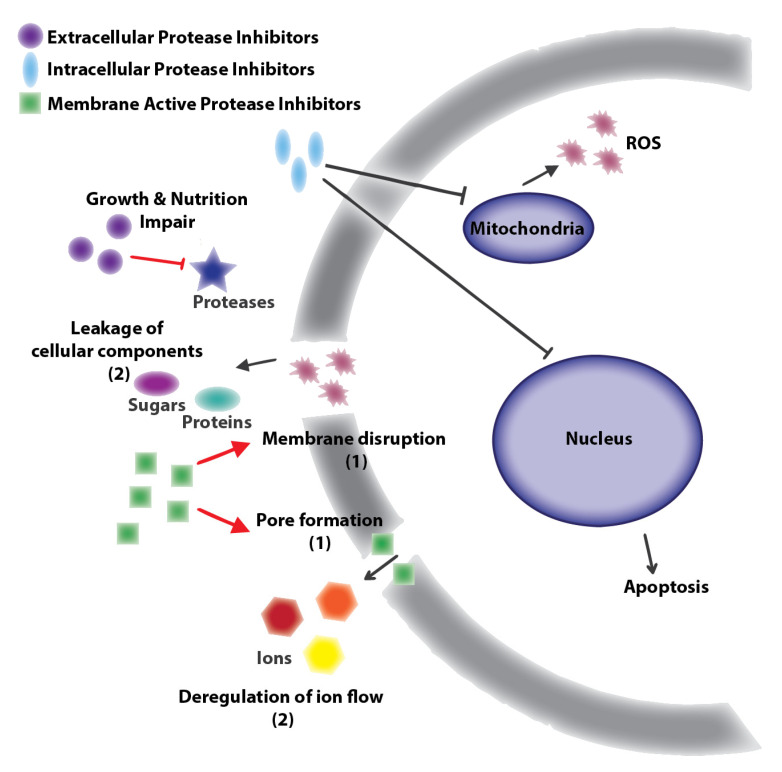
General targets of natural antifungal protease inhibitors: Protease inhibitors with extracellular targets produce nutrition or growth impairment by inhibition of nutrition related proteases [32,42,43,44]. Protease inhibitors with membrane cell targets cause disruption or pore formation leading to ion (e.g., Na^+^, K^+^, Ca^2+^ deregulation or leakage of cellular components [30,31]. Finally, protease inhibitors with intracellular targets inhibit mitochondria or nuclear proteases producing reactive oxygen species (ROS) or apoptosis [30,31]. Black lines correspond to antifungal compounds and red lines to molecules with similar antifungal or antibacterial effects.

Several natural protease inhibitors exert anti-virulence effects by targeting extracellular proteases, impairing nutritional and/or growth functions [30,31,32], or hindering virulence mechanisms, such as tissue invasion (Figure 1) [33]. For instance, secreted aspartic proteases (SAPs), are involved in several virulence processes, including tissue invasion, growth, and immune system evasion among the important human fungal pathogens, *Candida albicans* and *C. neoformans* [34,35]. Additionally, SAPs have been assessed as antifungal targets using protease inhibitors with promising results for further exploration [36,37,38]. Other important anti-virulence mechanisms include cell wall disruption or membrane pore formation initiated by protease inhibitors to deregulate ion flow and/or membrane disruption to cause leakage of internal cellular components, affecting cell viability [30,31]. Further, endogenous, or intracellular fungal proteases are involved in important mechanisms, such as protein maturation for development or growth, and apoptosis regulation [39,40] and natural protease inhibitors have reported intracellular targets (e.g., mitochondria or nucleus), producing damage by oxidative stress or apoptosis deregulation, affecting pathogen survival [30,31,32]. Notably, *C. neoformans* uses intracellular proteases for resistance against current antifungal treatments (e.g., site-2 protease), which is required for virulence and survival in the presence of azole drugs [41]. Therefore, compounds capable of crossing fungal membranes and inhibiting endogenous proteases constitute potential antifungal agents and perhaps opportunities to overcome resistance. However, it is important to note that evidence of targeting intracellular organelles of fungal pathogens also poses a risk of off-target effects with toxicity towards human cells. Therefore, investigation into precise mechanism(s) of action and targets is needed to assess the potential and requirement for inhibitor optimization. Recognizing the promise of targeting proteases with protease inhibitors for treatment of fungal pathogens, we continue with the description, identification, and examples of naturally-sourced protease inhibitors.

## 3. Classification of Naturally-Derived Protease Inhibitors

Naturally-derived protease inhibitors are generally small molecules (15 to 60 amino acids or organic compounds) and contain a relatively high content of disulfide bridges, conferring higher stability [45,46]. They are classified according to enzymatic specificity, such as serine, aspartic, or cysteine protease inhibitors [47], or according to structural features. For instance, natural protease inhibitors can be classified as Bowman–Birk serine protease inhibitors, which are typically 1.5 to 20 kDa with several sulfide bridges, commonly displaying specific activity towards elastase, trypsin, and chymotrypsin [48,49]. Kunitz-type inhibitors, which are low molecular weight proteins with two or three disulfide bridges and one reactive site, showing specificity towards serine proteases [50]. Another example includes Kazal-type inhibitors, which are double-headed and inhibit trypsin and chymotrypsin simultaneously [51,52]. Compared to chemically synthesized products, natural inhibitors are often designated as safer with a specific mechanism of action, which leads to fewer off-target effects. This is a desirable trait for the development of novel antifungals based on the close evolutionary relationship between fungi and the mammalian host [53,54]. Additionally, natural compounds have evolved to possess physiochemical properties, including the ability to penetrate bacterial cells, unlike synthetic molecules not subject to such evolution. Although bacterial and fungal cells are highly distinct (e.g., cell wall composition, presence of organelles), there are several reports of protease inhibitors with biological activity against both types of cells, suggesting that protease inhibitors with antibacterial activity have the potential for similar properties against fungal cells [44,53,55,56,57,58]. Furthermore, the evolution towards resistance against environmentally-sourced protease inhibitors is often reduced given the drive by natural selection to interact with cellular targets with high efficiency and selectivity to avoid resistance and off-target effects [59]. Based on the variety of potential targets of protease inhibitors, and advantages afforded by naturally-occurring protease inhibitors, we explore examples derived from plants, invertebrates, and microbes.

## 4. Plant-Derived Protease Inhibitors

Natural compounds produced by plants are an important source of bioactive molecules with a wide range of biologic targets, including protease inhibitors with regulatory roles for endogenous proteases, storage, and defense [60,61,62,63]. Over the last 20 years, the number of identified plant-derived protease inhibitors with anti-virulence activity has increased, corresponding with a heightened importance in biomedicine (Table 1). Here, we outline inhibitor activity and provide insight into mechanisms of action and potential roles against fungal pathogens.

### 4.1. Fabaceae (Leguminosae) Family

Kunitz-type trypsin inhibitors, ILTI and IETI, were isolated from seeds of the tropical trees, *Inga edulis* and *Inga laurica*, respectively [30,31]. These inhibitors have antifungal activity, showing growth inhibition towards *Candida tropicalis* and *Candida buinensis*. This activity is mediated by several mechanisms, including protease inhibition, alteration of the plasma membrane causing ion flow deregulation, triggering of oxidative stress by a mitochondrial target, or triggering of apoptosis in yeasts that block important serine peptidases (e.g., metacaspases), and a nuclear mediator of apoptosis (Nma111p) [40,67]. Similarly, the Kunitz-type trypsin and chymotrypsin inhibitors, ApTIA, ApTIB, and ApTIC, isolated from seeds of the Brazilian plant *Acacia plumosa* possess antifungal activity against *Aspergillus niger*, *Thielaviopsis paradoxa*, and *Colletotrichum* sp. P10 is associated with inhibition of serine proteases secreted by the fungi in growth medium, impairing nutritional mechanisms [32]. Another example includes the protease inhibitor, API, which is derived from the seeds *Albizia amara* Boiv., possessing antibacterial activity against *Pseudomonas aeruginosa* and *Bacillus subtilis* [55] and antifungal activity against several pathogens, such as *C. albicans* with a minimal inhibitory concentration (MIC) value of 32 µg/mL (comparable to current antimicrobials). Although, the target and mechanisms of API have not been reported, the observed inhibitory roles and relative potency support further exploration against additional fungal pathogens or investigation of synergistic activity with known antifungals.

Lastly, Lupinine and Diosgenin are two plant derived compounds that possess antifungal properties against *C. neoformans* [33]. These compounds inhibit a secreted metallopeptidase relevant in brain invasion by cryptococcal cells causing meningoencephalitis, CnMpr-1 (Inhibitory concentration [IC_50_] 5.025 µM and 9.659 µM, respectively) [68]. Lupinine is a quinolizidine alkaloid found primarily within flowering plants of the *Lupinus* genus [69], whereas diosgenin is a plant steroidal sapogenin isolated from dietary fenugreek (*Trigonella foenum-graecum*) seeds [70]. Interestingly, these compounds impair fungal crossing of the blood–brain barrier without detrimental effects to the host [33]. Diosgenin also inhibits matrix metalloproteinases (e.g., MMP-2 and MMP-9) involved in matrix integrity or cell migration [71,72,73,74,75]. Together, these compounds highlight the potential of plant-derived sources for inhibition of proteases produced by cryptococcal cells and underscores an opportunity for synergistic assessment with known antifungals and extrapolation to additional fungal pathogens.

### 4.2. Solanaceae Family

Potatoes (*Solanum tuberosum*) are a worldwide food staple; however, their global distribution also contributes to pathogen spread, affecting crop quality, and productivity. Defense proteins and peptides with antifungal and antibacterial activities derived from potatoes represent a reservoir for disease protection against both agricultural and medical pathogens [76]. For example, the peptide, Potide-G, isolated from the tubers of the potato *S. tuberosum* L. Cv. Golden Valley, is a Kunitz-type serine protease inhibitor that inhibits growth of diverse pathogens, including *C. albicans*, *Rhizoctonia solani*, *Staphylococcus aureus*, and *Listeria monocytogenes* through regulation of extracellular enzymes related to nutrition [44]. Potide-G possesses MIC values less than 30 µg/mL, a similar potency to other plant protease inhibitors and known antibiotics [44]. Similarly, PG-2, a peptide isolated from potato tubers of cv. Gogu Valley exhibits antifungal and antibacterial activity against *C. albicans*, *Clavibacter michiganensis* ssp. *michiganense*, and *S. aureus* [56]. In addition, PG-2 exerts minimal cytotoxic effects against human red blood cells, making the compound an interesting option for further investigation of direct and indirect targets. Other protease inhibitors derived from potato tubers include AFP-J, a serine protease inhibitor belonging to the Kunitz family isolated from cv. L. Jopung [64]. This protein inhibits chymotrypsin, pepsin, and trypsin, possessing antifungal activity against several microorganisms, including *C. albicans*, *Trichosporon beigelii*, and *Saccharomyces cerevisiae* with antimicrobial potency (MIC 6.25 µg/mL) like other antibiotics, and with no known hemolytic activity. To date, no direct target has been reported for this compound, and therefore, these results support further investigation to define the mechanisms of action and to uncover additional pathogenic targets.

### 4.3. Rhamnaceae Family

*Rhamnus frangula* is a tall deciduous shrub in the family *Rhamnaceae*. Crude extracts of *R. frangula* leaves exhibit antioxidant, antimicrobial, and free radical scavenging activities with a Kunitz-type serine protease inhibitor, RflP-1, isolated from leaves. This inhibitor acts on serine proteases of commercial fungal, such as *Aspergillus oryzae*, and bacterial proteases isolated from *B. licheniformis* [57,58]. RflP-1 also possesses an appreciable antibacterial action against both Gram-positive and Gram-negative bacteria with similar effectiveness of ampicillin [57]. However, no direct targets have been identified to date, supporting exploration to define the mechanism of action and potential extrapolation to other pathogens.

### 4.4. Rutaceae Family

*Clausena* is a genus comprising approximately 14 species of evergreen trees and the *Clausena lamsium* trypsin inhibitor, CLTI, is a homodimer isolated from the seeds that exerts anti-HIV activity (i.e., Anti-HIV-1 reverse transcriptase activity) and antifungal activity against *Physalospora piricola* [65]. Importantly, no molecular targets have been described for CLT1 to explain the antifungal activity and, considering the common co-infection of *C. neoformans* within HIV/AIDS patients, this protease inhibitor, and its derivatives show promise for synergistic antifungal properties.

### 4.5. Pinaceae Family

*Pinus* is a genus of vascular plants, commonly known as pines possessing abietic acid, an abietane diterpenoid found primarily in pine resin with inhibitory properties against *C. neoformans* by blocking crossing of the blood–brain barrier through CnMpr-1 inhibition (IC_50_ 5.143 µM) [33,77]. Similarly, some of the derivatives possess antimycotic and antibacterial activities [66], highlighting the potential of this compound as an important antifungal with broad reaching activity.

## 5. Invertebrate-Derived Protease Inhibitors

Invertebrates are a heterogeneous group of animals (about 1.3 million species) found ubiquitously within the environment, requiring strong defenses (e.g., production of chemicals) to adapt and survive against predators and pathogens, including protease inhibitors as self-defense systems [78,79]. For instance, many compounds with therapeutic potential detected from invertebrates show inhibition profiles against proteases with biotechnological and biomedical interest; although, many more remain to be studied [80,81,82,83]. Here, we present protease inhibitors derived from invertebrates and explore their described antimicrobial properties (Table 2).

### 5.1. Arthropoda Phylum

Within *Marsupenaeus japonicas* (a shrimp), a serpin type protease inhibitor, MjSerp1, exhibits inhibitory activity against microbial serine proteases, such as subtilisin A and proteinase K and, also inhibits the growth of Gram-positive (e.g., *S. aureus*, *B. subtilis*, and *Bacillus megaterium)* and Gram-negative bacteria (e.g., *Escherichia coli*, *Klebsiella pneumoniae*, and *Vibrio anguillarum*) [84]. Similarly found within this phylum, are the single WAP (whey acidic protein) domain (SWD)-containing protein, SWDPm2, which is a Type III crustin isolated from the black tiger shrimp, *Penaeus monodon* [85]. This molecule is a potent competitive-type inhibitor of subtilisin A, a typical member of the S8 family, which is widely distributed among all kingdoms and in several human pathogens [88]. The primary functions of SWDPm2 include antimicrobial action and inhibition of bacterial peptidase to limit microbial infection and pathogenesis, as well as antibacterial activity against several Gram-positive bacteria (e.g., *S. aureus*, *Aerococcus viridans*, and *B. megaterium*). Although a mechanism of action remains to be defined, a potential for antifungal activity represents a new avenue of study as some human fungal pathogens, such as *C. neoformans* also use extracellular subtilisin-like proteases in their pathogenic mechanisms (e.g., Cerevisin and Pqp1) [35,89,90]. Lastly, BmoSPI51 is Kunitz-type trypsin inhibitor isolated from silkworm (*Bombyx mori*) cocoon with inhibitory growth properties against fungi, including *S. cerevisiae* and *C. albicans* [86,91]. Following fungal infection, BmoSPI51 production increases in *B. mori* supporting a role in immunity, such as protecting silk fibroin proteins from degradation by fungal enzymes [92]. Additionally, approximately 80 potential protease inhibitors from several families (e.g., TIL-type, Kunitz-type, and Kazal inhibitors) have been reported in the silkworm using genomic approaches [93], highlighting this organism as a rich source of new protease inhibitors with potential antifungal properties.

### 5.2. Mollusk Phylum

Mollusks present a wealth of natural compounds displaying antimicrobial activity, including 19 within the global marine pharmaceutical clinical pipeline and four approved by the US Food and Drug Administration to date [94]. Notably, over half of the secondary metabolites produced by mollusks have yet to be evaluated for bioactivity, representing a plethora of new avenues to pursue for in vitro, in vivo, and clinical studies [83,95]. For instance, protease inhibitors have been reported from oysters, such as *Crassostrea gigas* peptides, which are competitive inhibitors of HIV-1 protease with an inhibitory constant (k_i_) between 10 and 13 nM [87]. Inhibitory potency of these compounds is like the first generation of synthetic HIV-1 protease inhibitors, such as Indinavir, but lower than second generation options, such as Atazanavir (k_i_ = 10 pM) [96,97]. Although the potency of these peptides can be improved through development and optimization of synthetic versions, the initial discovery and activity of naturally-produced compounds from mollusks shows great promise for new avenues of exploration. Furthermore, several HIV-1 protease inhibitors possess antifungal activity, mainly through inhibition of SAPs [98,99,100]; highlighting the potential of these peptides as future antifungal compounds and warranting further investigation.

## 6. Bacterial Protease Inhibitors

Protease inhibitors produced by microorganisms have protective roles against endogenous proteases. Conversely, secreted microbial protease inhibitors may modulate external proteolytic degradation to benefit the producer. For example, a microbe may secrete a protease inhibitor to regulate their own bacterial proteases (i.e., self-defense), defend against other microbes and infections, protect from predation, or in response to host proteases produced during invasion. Due to the importance of protease inhibitors produced by bacterial species, they have been extensively studied with the intent for developing novel therapeutic drugs [101,102]. Here, we highlight bacterial sources of protease inhibitors and discuss their relevance as antimicrobial strategies against other pathogens (Table 3).

### 6.1. Actinomycetaceae Family

Pepstatin A is a microbial hexapeptide produced by *Actinomycetes* spp. and a potent inhibitor of almost all types of aspartic proteases, including SAPs [104]. This inhibitor modulates virulence of the SAP family and inhibits cell proliferation and adhesion to abiotic and biotic structures of *Candida* spp. showing promise as an antifungal therapeutic [42]. However, when administered intravenously, pepstatin A is ineffective in systemic infections, due to its unfavorable pharmacokinetic properties, underscoring the relevance and need for optimization [104,105,106]. Structural modifications may, therefore, present an opportunity for the design of novel potent and SAP inhibitors with antifungal properties.

### 6.2. Bacillaceae Family

Isolated from the extremophile *Bacillus* spp., ATBI, is a peptide and potent inhibitor of several aspartic proteases, including recombinant HIV-1 protease, pepsin, and fungal *Aspergillus saitoi* (F-Prot) aspartic protease [103]. ATBI binds within the active site of the HIV-1 protease (competitive inhibition), leading to inactivation of the enzyme, and thereby suggesting pharmaceutical potential as a drug for the treatment of AIDS. As described above, compounds such as HIV-1-protease inhibitors (e.g., indinavir or ritonavir) possess antifungal properties [36,37,38,99], highlighting the need for more research using ATBI against human fungal pathogens, such as *Candida* spp. and *C. neoformans*.

## 7. Future Directions and Conclusions

Our presentation of representative protease inhibitors derived from natural sources, including plants, invertebrates, and microbes underscores the immense potential of not only identifying and characterizing new natural compounds from these sources and others, but also, outlines opportunities for synthetic compound design based on informed observations. As identified here, important areas for further exploration include the search for natural compounds that mimic current synthetic compounds with anti-HIV activity. For example, the beneficial effects of anti-HIV protease inhibitors on the incidence of disease and the subsequent outcome of opportunistic fungal infections, such as candidiasis [36,37,98] and cryptococcosis [38,99,100,107,108]. This includes the off-target effects of anti-HIV aspartic protease inhibitors (e.g., saquinavir, indinavir and ritonavir) against hydrolytic enzymes (e.g., SAPs in *C. albicans*), which correspond with reduced fungal infections in HIV-infected patients [36,37,98,109]. Additionally, the HIV aspartic protease inhibitor, indinavir, selectively inhibits the production of proteases and urease by *C. neoformans*, interfering with capsule formation and resulting in heightened susceptibility of fungal cells to intracellular killing by natural effector cells [99]. In addition, prolonged incubation of *C. neoformans* with indinavir inhibits fungal growth, reducing virulence, and enhancing susceptibility to the endogenous antimicrobial activity of natural effector cells [108]. These unintended benefits of treating a viral infection led to increased host response and protection against fungal infections. Another avenue includes extrapolating the success of anti-bacterial protease inhibitors towards fungal proteases. For instance, the *Euphorbiaceae* family non-competitive trypsin inhibitor, JcTI-I demonstrates inhibitory activity against proteases from *S. aureus* and *Salmonella enteric* [43]. This inhibition is with high potency and low cytotoxicity making JcTI-I a pharmacologically interesting and valuable drug for the design of a novel antibiotic, but observations against fungal pathogens have not been reported [43].

Over the last 20 years, natural protease inhibitors and their biological activities have been reported from diverse sources (see Table 1, Table 2 and Table 3) with plants and invertebrates being rich reservoirs of compounds with biomedical applications. However, the isolation of microbes from the environment fails to capture the relationships among microbes within that environment (e.g., soil microbiome). Such interactions may drastically alter the production and abundance of proteins, as well as differences in protein profiles conveyed by microbes in the laboratory vs. the natural setting. This makes a comprehensive identification and appreciation of the intricacies of microbe–microbe interactions nearly impossible to replicate, suggesting that our current observations and discoveries are incomplete. Moreover, the selection criteria within clinical trials for efficacy, bioavailability, resistance, safety, and cost are critical to monitor and assess potential harmful outcomes for the host [29,96]. Considering the need for balanced specificity, which promotes potency of the inhibitor but allows for potential off-target effects, beneficial properties not previously anticipated (e.g., anti-HIV protease inhibitors described above) could therefore, be uncovered.

As emphasized in this Review, the potential for exploration of diverse protease inhibitors against fungal pathogens exposes our limited knowledge of defined mechanisms of antifungal activity. This Review provides insight into selectivity and off-target effects to move the described in vitro studies from the lab bench and into the clinic [110]. Finally, while we focus on protease inhibitors with relevance against biomedical fungal pathogens, opportunities and applications presented in this Review extend, through crosstalk and cross-reactivity of protease inhibitors, to the plethora of fungal pathogens currently impacting the agricultural sector and threatening global food security.

## Figures and Tables

**Table 1:** Protease inhibitors derived from plants with antimicrobial activity.

Source	Protease Inhibitor Designation (Source)	Enzymatic Family	MW (kDa)	Activity (Mechanism of Action)	Reference
*Fabaceae* *(Leguminosae)*	IETI (*Inga edulis*)	Kunitz	19.7	Antifungal (Protease inhibition, membrane disruption and oxidative stress)	[31]
	ILTI (*Inga laurica*)		20		[30]
	ApTI (A, B, C) (*Acacia plumosa*)	Kunitz	20	Antifungal (Secreted protease inhibition and nutrition impairment)	[32]
	API (*Albizia amara*)	Unknown	49	Antifungal and Antibacterial	[55]
	Lupinine (*Lupinus* spp.)	Quinolizidine alkaloid	0.17	Anticryptococcal (secreted metallopeptidase inhibition)	[33]
	Diosgenin (*Trigonella foenum-graecum*)	Steroidal sapogenin	0.41		
*Solanaceae*	Potide-G (*S. tuberosum* L. Cv. Golden Valley)	Kunitz	5.57	Antibacterial and Antifungal (Secreted protease inhibition and nutrition impairment)	[44]
	PG-2 (*S. tuberosum* L. Cv. Gogu Valley)	Kunitz	3.2	Antibacterial and Antifungal	[56]
	AFP-J (*S. tuberosum* L. Cv. L. Jopung)	Kunitz	13.5	Antifungal	[64]
*Rhamnaceae*	RflP-1 (*Rhamnus frangula*)	Kunitz	22.5	Antibacterial and Antifungal	[57,58]
*Rutaceae*	CLTI *(Clausena lamsium)*	Unknown	54	Anti-HIV-1 reverse transcriptase activity and Antifungal	[65]
*Pinaceae*	Abietic acid *(Pinus* spp.)	Abietane diterpenoid	0.3	Anticryptococcal (secreted metallopeptidase inhibition)	[33,66]

MW: Molecular weight.

**Table 2:** Protease inhibitors derived from invertebrates with antimicrobial activity.

Source	Protease Inhibitor Designation (Source)	Family/Chemical Class	MW (kDa)	Activity (Mechanism of Action)	Reference
*Arthropoda*	MjSerp1 (*Marsupenaeus japonicas*)	Serpin	46.3	Antibacterial	[84]
	SWDPm2 (*Penaeus monodon*)	Type III crustin	7.38		[85]
	BmoSPI51 (*Bombyx mori*)	Kunitz-type	14	Antifungal	[86]
*Mollusk*	Peptides (*Crassostrea gigas*)	Unknown	Unknown	HIV protease inhibitor (Competitive inhibition)	[87]

MW: Molecular weight.

**Table 3:** Protease inhibitors derived from bacteria with antimicrobial activity.

Source	Protease Inhibitor Designation (Source)	Family/Chemical Class	MW (kDa)	Activity (Mechanism of Action)	Ref.
*Actinomycetaceae*	Pepstatin A (*Actinomycetes* spp.)	Hexapeptide	0.68	Antifungal (Secreted protease inhibition) and HIV protease inhibitor	[42]
*Bacillaceae*	ATBI (*Bacillus* spp.)	Heptapeptide	1.1	HIV-1 protease inhibitor (Competitive inhibition)	[103]

MW: Molecular weight.

## Data Availability

Not applicable.
